# Digital acoustofluidics enables contactless and programmable liquid handling

**DOI:** 10.1038/s41467-018-05297-z

**Published:** 2018-07-26

**Authors:** Steven Peiran Zhang, James Lata, Chuyi Chen, John Mai, Feng Guo, Zhenhua Tian, Liqiang Ren, Zhangming Mao, Po-Hsun Huang, Peng Li, Shujie Yang, Tony Jun Huang

**Affiliations:** 1 Department of Mechanical Engineering and Material Science, Duke University, Durham, NC 27708 USA; 2 Department of Engineering Science and Mechanics, The Pennsylvania State University, University Park, State College, PA 16802 USA; 3 Alfred E. Mann Institute for Biomedical Engineering, University of Southern California, Los Angeles, CA 90007 USA

## Abstract

For decades, scientists have pursued the goal of performing automated reactions in a compact fluid processor with minimal human intervention. Most advanced fluidic handling technologies (e.g., microfluidic chips and micro-well plates) lack fluid rewritability, and the associated benefits of multi-path routing and re-programmability, due to surface-adsorption-induced contamination on contacting structures. This limits their processing speed and the complexity of reaction test matrices. We present a contactless droplet transport and processing technique called digital acoustofluidics which dynamically manipulates droplets with volumes from 1 nL to 100 µL along any planar axis via acoustic-streaming-induced hydrodynamic traps, all in a contamination-free (lower than 10^−10^% diffusion into the fluorinated carrier oil layer) and biocompatible (99.2% cell viability) manner. Hence, digital acoustofluidics can execute reactions on overlapping, non-contaminated, fluidic paths and can scale to perform massive interaction matrices within a single device.

## Abstract (web summary)

Contamination is an obstacle to the functioning of microfluidic devices. Here the authors exploit acoustic streaming to manipulate droplets which float on a layer of immiscible oil. This prevents contamination and enables rewritability by which different fluids can be used on the same substrate.

## Introduction

Handling of liquids is essential in a majority of applications associated with chemical, biological, and biomedical protocols. Current automated fluid processing technologies^1–4^ have brought unprecedented accuracy, speed, and repeatability to biomedical research and the pharmaceutical industry, such as the preparation of sequencing libraries, clinical diagnostics, and large-scale compound screening. A myriad of different liquid handling mechanisms, including robotics^4^, micro-droplets^5–7^, pneumatic valves^8–10^, electrical^11–17^, acoustics^18–26^, hydrodynamics^27–31^, magnetics^32^, and liquid marbles^33^, have been applied to numerous fluid processing scenarios. Among all these mechanisms, the robotic pipetting system (e.g., Cloud Lab^4^) is, by far, the most widely adopted solution for automation, yet it requires considerable expense, space, and maintenance. Lab-on-a-chip solutions miniaturize the bulky wet lab onto a compact chip^2^, but are generally optimized for specific reaction protocols and hence lack programmability and dynamic re-configurability since all channel designs are fixed after fabrication. Digital microfluidics offers an appealing solution for efficient automation by programmable manipulation of nano- to pico-liter droplets on a miniaturized device using electro-wetting forces^11–16^. It has demonstrated remarkable programmability in terms of reaction automation, particularly for reaction protocols that require serial addition of reagents or precise temporal control.

Despite these strengths, most current methods suffer from a fundamental constraint. They generally rely on physical contact with a solid structure in order to contain, transport, or manipulate liquid reagents. Therefore, traces of a reagent inevitably adsorb onto the contact surface and can possibly later dissolve into another liquid sample. Thus, the risk of cross-contamination due to this undesirable “fouling of the surface”^34^ intrinsically limits the transport surfaces to a single type of working liquid plus reagent combination, and restricts the repetitive actuation of liquids with sticky biomolecules (e.g., undiluted blood). So a matrix of successive cascading interaction experiments requires an exponential number of available channel paths, as well as a complex, multi-layered array of independently-actuated switches, to avoid cross contamination.

Contactless liquid-handling methods, in contrast, eliminate surface adsorption by employing long-range forces to isolate liquid reagents from solid structures, allowing for massively scalable, dynamic, multi-path fluidic processing due to the custom programmable and rewritable nature of the fluidic channels. The rewritability (i.e., the ability to reuse the same fluidic path without cross contamination) enables the use of multi-path routing and test optimization with respect to time and space when applied to the testing of large matrices of experimental variables. We label this advantage as “droplet rewritability” since there can be many different possible reagent combinations within a droplet which is enabled by reusable paths for transportation or mixing, even with a small array of acoustic transducers. Recently, there has been renewed interest in acoustics as a straightforward and promising solution for liquid handling owing to its contactless operation, label-free selectivity, and high biocompatibility^35–39^. An embodiment of contactless, acoustic-based liquid handling was in the form of ultrasonic levitation^19^. This method used a standing bulk acoustic wave to suspend liquid drops at pressure nodes in open air. These acoustic mechanisms have evolved to either holographic^40–42^ or digital^43^ manipulation of levitated objects or droplets. However, with its limited resolution, low controllability, and bulky transducer size, ultrasonic levitation is not suitable for manipulation in small scales. Not only does ultrasonic levitation have difficulties in accurately manipulating small objects (e.g., microparticles or nanoliter droplets), it also presents significant challenges when trying to load or collect these objects from the device.

Herein we present digital acoustofluidics, an acoustic-based, programmable, contactless liquid-handling technology, which allows one to digitally transport, merge, mix, and split reagents within aqueous droplets in a contamination-free, biocompatible manner. In digital acoustofluidics, the droplets are floating on an inert, immiscible layer of oil that effectively isolates the droplet above a solid surface. We demonstrate fluidic manipulation between potential wells, characterize the acoustic streaming within and around the droplets, integrate the on-demand traps into a digital fluidic processor, evaluate the risk of cross-contamination, and finally apply digital acoustofluidics using six droplets to perform an optimized cascading sequence of enzymatic reactions. In comparison to existing fluidic processing approaches, the digital acoustofluidics platform has four advantages as rewritability (i.e., reusable fluid-paths), biocompatibility (e.g., 99.2% Hela cell viability), versatility (i.e., suitable for handling a wide range of liquids and solids, such as organic solvents, blood, sputum, reactive fluids, and fecal samples), and uniformity (i.e., minimizing internal polarization in aqueous droplets). Thus digital acoustofluidics offers unique pathways for addressing the obstacles in existing liquid-handling systems associated with surface adsorption, surface degradation, and internal polarization. It provides a compelling platform for the development of robust, rewritable, and digitally programmable fluidic processors.

## Results

### Three-dimensional acoustic streaming induces hydrodynamic traps

**Fig. 1:**
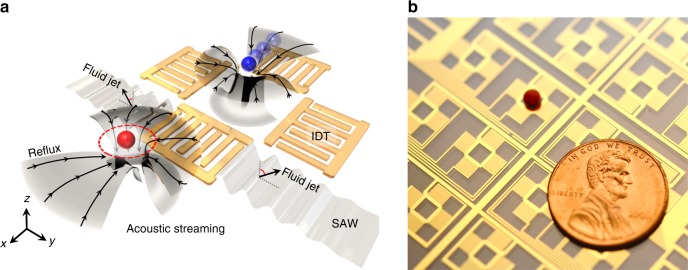
Digital acoustofluidics for contactless and programmable droplet manipulation. **a** Schematic showing one unit consisting of four IDTs (one pixel) in the digital acoustofluidic device. The four IDTs can be selectively excited to translate droplets along the ±*x* and ±*y* directions. The aqueous droplets are isolated from the piezoelectric substrate by an inert carrier fluid to prevent direct contact with surfaces. The IDT (bottom left) embedded beneath the carrier fluid generates SAWs that pumps out fluid in the ±*y* directions and pumps in fluid in the ±*x* directions. The red and blue droplets are separately trapped at the two symmetric hydrodynamic wells near the flanks of an IDT. The blue droplet is translated toward a well on the other side of the excited transducer. The reflux streamlines are shown in black. **b** A photo showing the digital acoustofluidic device with a drop of blood floating on the carrier layer of fluorinated oil

As shown in Fig. 1a, interdigital transducers (IDTs) fabricated on a LiNbO_3_ substrate are employed for generating acoustic waves. Each array of four IDTs can be considered as a single pixel, a fluidic “step” for transport or as a processing site.

In order to manipulate aqueous droplets along a horizontal plane without direct contact with the surface, a denser carrier layer of fluorinated oil is added to the LiNbO_3_ substrate, not only as an isolation layer upon which the droplets float, but also as an actuator to drive droplets via the drag force induced by acoustic streaming. For an immersed IDT, its excitation force directly loads on the carrier oil layer above the IDT and generates bulk waves in the oil layer. Meanwhile, surface acoustic waves (SAWs) are also generated, and these propagate along the substrate surface and leak into the carrier oil as leaky SAWs. The contributions of both bulk waves and leaky SAWs actively create two symmetric fluid jets out of the substrate plane, in the oil layer (Supplementary Fig. 1). The measured jetting angle with respect to the IDT apertures is 34° (Supplementary Fig. 2), which matches with the Rayleigh wave mode on the Y-128^o^ cut LiNbO_3_ substrate (longitudinal-to-transverse amplitude ratio of 0.7^44^). Those two symmetric fluid jets impinge on the air-liquid interface, recirculate back, interact with each other and the bottom boundary, and finally complete a butterfly-wing-shaped streaming pattern in the far field (Supplementary Fig. 3),^45^ as well as two localized symmetric hydrodynamic traps near the flanks of the transducer (Fig. 1a). In other words, the IDT acts as a micropump that pushes fluid out along the ±*y* directions and pumps fluid in along the ±*x* directions (Fig. 1a). Hence, in a digital acoustofluidic device (Fig. 1b), a droplet floating on the oil surface will be driven toward the sides of the IDT in the ±*x* directions by the drag force from the oil being pumped in.

**Fig. 2:**
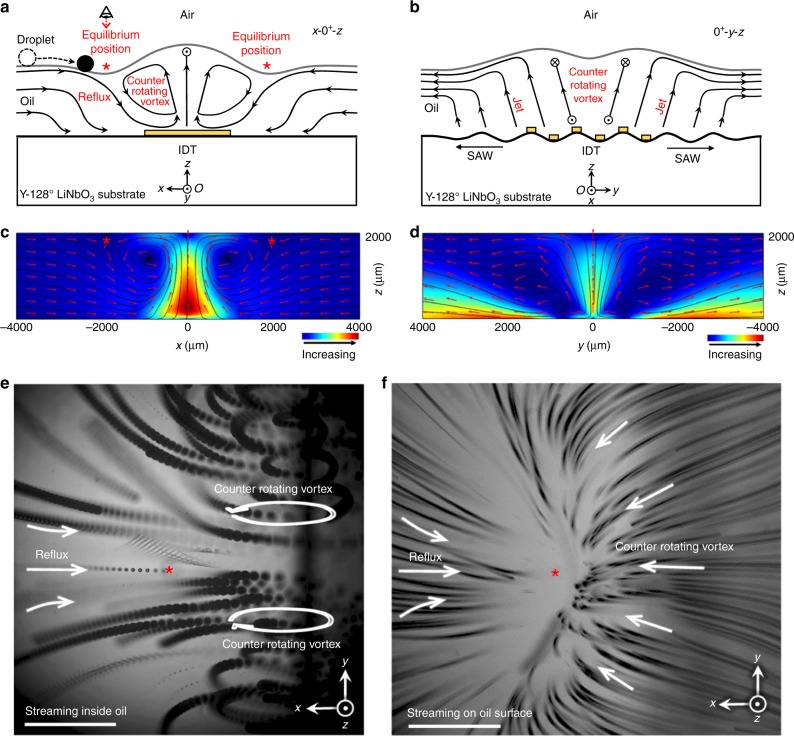
Mechanism of hydrodynamic traps. **a** *x–z*-plane cross-sectional view of the streaming pattern at an IDT (at *y* = 0^+^). The IDT pumps out the carrier fluid in ±*x* directions. The origin (*O*) is located at the center of the IDT on the *x–y*-plane. The reflux pulls the droplet toward the IDT in the far field, and then the counter-rotating vortex near the side of the IDT resists this reflux and finally stabilizes the droplet. The red asterisks indicate the symmetric hydrodynamic equilibrium positions on the surface of the carrier oil. **b** *y–z*-plane cross-sections of the streaming pattern at an IDT (at *x* = 0^+^). The IDT pumps in the carrier fluid in the ±*y* directions. The waves propagating around the carrier fluid generated two symmetric jet flows with an incident angle of 34° with respect to the IDT aperture. **c** Simulation results showing the acoustic streaming patterns in the *x–z*-plane. The counter-rotating vortices resist the reflux and form two hydrodynamic traps near the sides of the transducer (as indicated by the red asterisks). **d** Simulation results showing the acoustic streaming patterns in the *y–z*-plane. The acoustic energy from the IDT coupled into the oil layer above it and pumped out fluid in the ±*y* directions. **e**, **f** Stacked images of particle trajectories near one flank of an activated IDT. This region corresponds to the area highlighted by the red circle in Fig. 1a. The reference perspective is also indicated by the eye symbol in **a**. **e** *z* = 1.3 mm, the focal plane is in the middle of the carrier fluid layer. In this composite image, the reflux and the vortices of the counter flow are clearly visualized. The red asterisk indicates the hydrodynamic equilibrium position on the surface of the carrier layer of oil. **f** *z* = 2 mm, the focal plane of the microscope is fixed to the surface of the oil layer. The counter-rotating vortices form a clamp-like pattern which pinches the droplet and keeps it from escaping in +*y* and −*y* directions. Scale bar: 750 μm

Figure 2a, b schematically shows cross-sections of streamlines in the *x–z-* and *y–z-*planes, with the corresponding numerical simulation results given in Fig. 2c, d. Specifically, the floating droplet near a side of the IDT will be automatically translated in the ±*x* directions toward the IDT following a hydrodynamic gradient. Eventually, the droplet is stabilized at one of the hydrodynamic equilibrium positions due to the force balance in the *x* directions between the ‘reflux’ from the far field and the two counter-rotating vortices near the flanks of the transducer (Fig. 2a, e). The streamlines from these two counter-rotating vortices gradually extend in the +*y* and −*y* directions and then recirculate back, finally forming a three-dimensional clamp-like shape (Fig. 2f) near the surface of the oil, which traps the droplets from escaping in the +*y* or −*y* directions.

During the droplet-trapping process, the far field reflux first pulls the droplet toward the transducer, then the counter-rotating vortex resists the reflux and finally pinches the droplet at one of the flanks of transducer. The hydrodynamic potential well on the surface of the oil is clearly visualized in the image of the particle trajectories (Fig. 2f). Once a potential well is occupied by a droplet, the streamline pattern in the oil layer equilibrates spontaneously, yet maintains a similar pattern (Supplementary Fig. 4).

### Droplet trapping via acoustic streaming

**Fig. 3:**
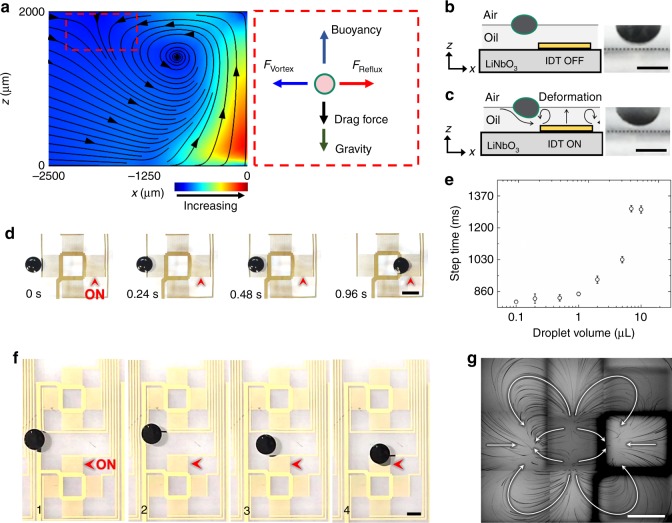
Characterization of the hydrodynamic trap. **a** Calculated streamlines from a numerical model of the forces generated by acoustic waves in the *x*–*z*-plane, and an illustration of the force balance along the *x*–*z-*plane at the trap. The counter-rotating vortices are clearly seen. Reflux and counter-rotating vortices cancel each other out and stabilized the droplet in the ±*x* directions. The drag force represents the sum of *z*-components of drag forces from counter-rotating vortex and reflux. **b** Side view of the immersed part of a 5 µL droplet when the IDT is turned off. The gray dashed line indicates the plane of the LiNbO_3_ substrate. **c** Side view of the immersed part of a 5 µL droplet when the IDT is turned on. The droplet slightly deforms upon the activation of acoustic streaming but remains floating on the oil layer, and above the substrate. **d** A sequence of time-elapsed, top-view images of the droplet-trapping process. The red arrow indicates the activated IDT. Two symmetrical hydrodynamic traps are created on opposite sides of the transducer, and the nearby droplet is transported by following the hydrodynamic gradient. Note that at 960 ms, the trapped droplet is mildly pinched and deformed by the counter-rotating vortices near the two transducer apertures. The 5 µL droplet’s shape conforms with the streamline distribution in Fig. 2f. **e** The relationship between the droplet volume and the step time. ‘Step time’ represents the time needed for translating 6.5 mm and stopping a droplet, the distance of a single step. **f** A free-floating droplet is restabilized near an excited IDT. The droplet naturally follows the local pressure gradient towards the IDT and then is stabilized at one of its hydrodynamic traps. **g** Particle tracking result showing the streaming pattern on the oil’s surface (top view). All streamlines of the reflux and the counter-rotating vortices converged at the two hydrodynamic traps. For a more detailed description of this image, please see Supplementary Fig. 3. All scale bars: 2 mm

An illustration of the force balance on a trapped droplet is shown in Fig. 3a. Before trapping, the free-floating droplet has a flat-shaped geometry due to the balance between the forces of gravity, buoyancy, and surface tension (Fig. 3b). Once trapped, the droplet is dragged down and is slightly deformed (Fig. 3c) mainly by the streaming, but still is not in direct contact with the substrate. The experimental droplet-trapping process is shown in Fig. 3d. The droplet is first pulled toward an excited IDT (indicated by the black arrow) and then is stabilized directly above the left flank of IDT. In the far field, with respect to the excited IDT, the reflux dominates and gradually pulls the droplet towards the hydrodynamic equilibrium position (Supplementary Fig. 5, 0 ~ 8.5 mm). As the droplet approaches the hydrodynamic equilibrium position, the opposing force from the counter-rotating vortices increases sharply (Supplementary Fig. 5, 8.5 ~ 9 mm) and finally balances with the pulling force from the reflux. As a result, the droplet accelerates gradually in the first 8.5 mm and then drastically decelerates to a static state within the next 0.5 mm. The dependence between the droplet volume and a given step time (e.g., the travel time for a droplet to traverse a single step of 6.5 mm) is shown in Fig. 3e. The step time increases from 819 to 1337 ms as the droplet volume increases from 0.1 to 10 µL. The resolution of the digital acoustofluidic device for a single planar translation is determined by the spacing between two adjacent IDTs. For example, for the chip shown in Fig. 1b, the spacing between two adjacent IDTs is 6.5 mm. The minimum translation distance between two adjacent IDTs is 1.0 mm, as shown in Supplementary Fig. 6. Notably, even for a droplet that is not initially well-aligned with the transducer, it will still be gradually re-aligned by the hydrodynamic gradient and eventually be trapped by the potential well (Fig. 3f), which is consistent with the description of the streaming pattern on the surface of the oil (Fig. 3g).

**Fig. 4:**
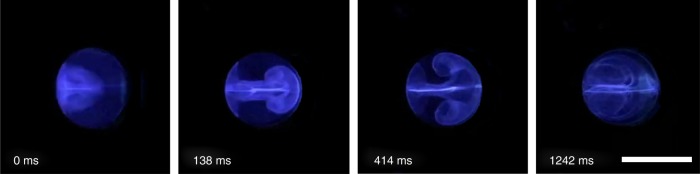
Internal streaming within a droplet. A trapped 50 µL droplet with super-signal ELISA luminescent substrate is merged with a droplet containing horseradish peroxidase (HRP). Two symmetric vortices are visualized by the luminescence emitted upon oxidization of the luminol substrate. The luminol-containing droplet was first stabilized at one-pixel unit and then merged with a HRP-containing droplet. Luminescence was emitted instantly upon merging. Digital acoustofluidics generates strong internal fluidic streaming within the trapped droplets, thereby minimizing internal polarization in aqueous droplets and enabling efficient mixing of merged droplets. Supplementary Movie 1 demonstrates the dynamic process of inner streaming. Scale bar: 5 mm

Aside from the movement of the droplet, two symmetric vortices (internal streaming) are also induced inside of the trapped droplet (Fig. 4), due to momentum continuity at the immiscible interface between the aqueous droplet and the carrier oil. These internal vortices enable rapid and uniform mixing (shorter than 216 ms for complete mixing) and the detachment of particles from the polar–non-polar interface at the water-and-oil boundary.

### Digitally programming droplets

**Fig. 5:**
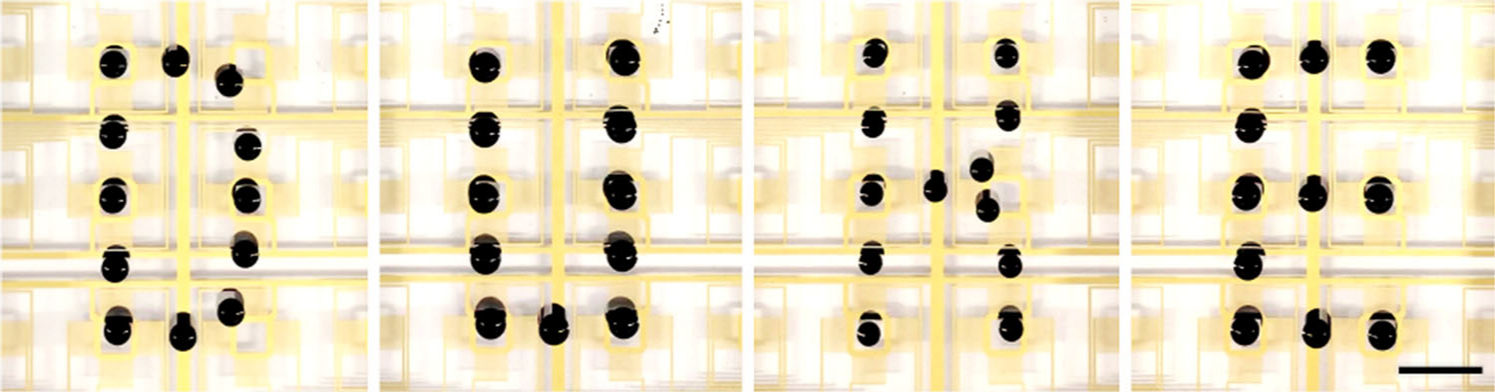
Programmable single-droplet transport via digital acoustofluidics. The hydrodynamic traps are activated following a pre-programmed movement sequence, resulting in the digital hydroacoustic formation of the letters ‘DUKE’. Each pixel consists of four independent IDTs; therefore, each droplet can be transported dynamically in any direction on the *x*–*y*-plane using a trap-and-release mechanism using an off-chip electromechanical relay controller. Scale bar: 8 mm

By arranging the IDTs into an 8 × 8 array on a LiNbO_3_ wafer, a programmable microfluidic processor with 16 individual pixel units has been developed. By selectively activating the nearest IDT in adjacent pixel units, each droplet can be individually *x*- or *y*-translated from pixel to pixel following the triggering of the appropriate hydrodynamic gradients (Supplementary Fig. 7). Based on this principle, sophisticated manipulation and routing for the droplets is achieved (Fig. 5). Furthermore, due to the contact-free manipulation feature, the acoustic transducers require no continuous surface and can be discretized into multiple individual transducers that are compatible with a printed circuit board (PCB) (Supplementary Fig. 8) for easier re-configurability and simpler electric connections.

**Fig. 6:**
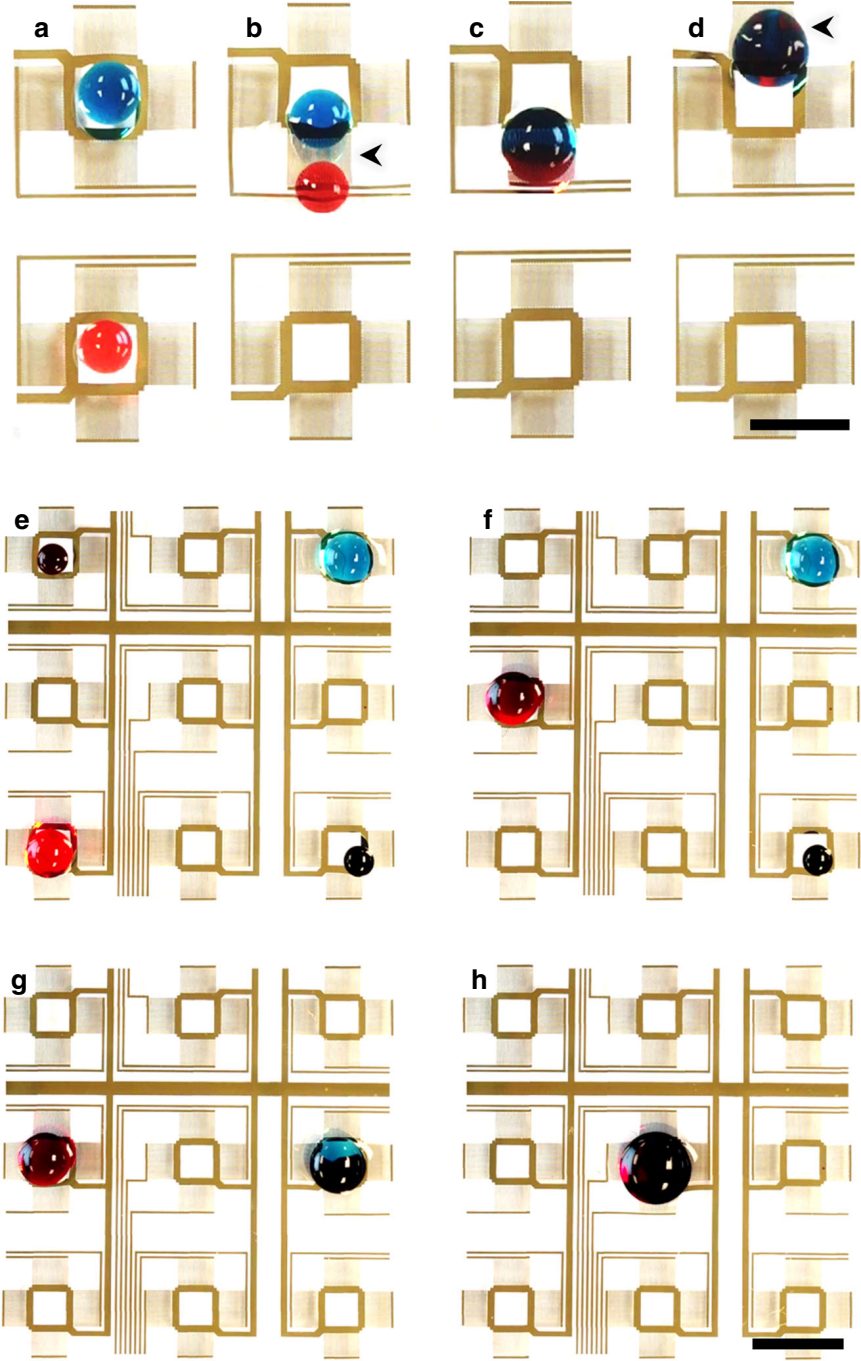
Droplet merging using a digital acoustofluidic device. **a**–**d** The merging process of two droplets. Scale bar: 5 mm. The black arrows indicate the activated transducers. Supplementary Movie 2 shows the complete merging process. **e**–**h** Two-stage sequential merging of four dye-containing droplets. The first-stage brings the vertically aligned droplets together along the center line where they are then horizontally combined for the second-stage reaction at the center pixel. Scale bar: 8 mm

Droplet fusion is a major functional capability in this digital acoustofluidic device which enables automating an entire reaction test matrix. Briefly, two droplets are first transported into two neighboring pixels. They are then moved and localized within the two symmetric hydrodynamic traps generated on each side of a single IDT (Fig. 6a). Once the IDT is deactivated and the hydrodynamic traps vanished, the two droplets move towards each other and merge following the Cheerios effect^46^ (Supplementary Movie 2). In addition to the fusion of two droplets, we also demonstrate a programmed multi-step reaction, which is crucial for reactions that require the serial addition of reagents. For visual clarity, two black colored droplets are separately first merged with blue and red droplets, respectively. Then these two product droplets are moved and are merged with each other (Fig. 6b).

With this digital acoustofluidic system, not only can we manipulate microliter-scale droplets, but we can also manipulate microparticles (Supplementary Fig. 6), nanoliter-scale droplets (Supplementary Movie 3–5), and even sub-nanoliter droplets (Supplementary Fig. 9) by further minimizing the droplet dimensions and optimizing the arrangement of transducers (e.g., IDT dimension: 600 × 400 µm, frequency: 49.125 MHz, voltage: 5.0‒7.0 Vpp). Successful manipulation of droplets with over a 1000 times difference in volumes reveals the potential of our digital acoustofluidic system for multi-scale integration as well as massively parallel processing of test matrices.

Supplementary Fig. 10 and Supplementary Movie 6 show acoustofluidic-based on-demand droplet generation. Supplementary Fig. 11a shows the interface between the acoustofluidic droplet generator and the digital acoustofluidic chip. The generated droplet floated to the surface of oil, and then was manipulated by the digital acoustofluidic chip (Supplementary Fig. 11b‒h). Droplet splitting via acoustic streaming is preliminarily achieved with a droplet floating directly above the geometric center of an IDT. As shown in Supplementary Fig. 12a, the IDT is excited at a high voltage (i.e., 150 Vpp) and forms an ascending flow-jet in the center as well as two symmetric hydrodynamic traps on both flanks of IDT. A droplet positioned directly above the geometric center of IDT will be halved into two smaller droplets. Supplementary Fig. 12b‒h and Supplementary Movie 7 show the combined droplet transportation and splitting process.

### Characterization of cross-contamination level

Fluorinert FC-70 is a fluorinated oil and is immiscible in water, chemically inert, and has been widely applied in droplet microfluidics and liquid breathing experiments due to these properties and its oxygen-permeability^47^. In our configuration, since there is no direct liquid-solid contact by the droplet, the dominant potential source of cross-contamination comes from the diffusion of reagents. According to a previous diffusion study in fluorinerts^48^, we carefully selected fluorescein and Rhodamine 6G as the indicators to characterize the diffusion. The experimental details are described in Methods section. After an 8-h co-incubation experiment (100 µM dye solution and oil) at 55 ℃, the detected diffusion of fluorescein in FC-70 oil was below 10^−10^ µM (lower than 10^−10^%), indicating that any fluorescein which diffused into the fluorinert was below the detection limit of the plate reader. Although Rhodamine 6G diffused more effectively than fluorescein in the fluorinated oil, the oil showed excellent resistance to diffusion within 4 h at 20–55 ℃ (lower than 10^−10^%). This diffusion value is equivalent to, or lower than, the natural fluorescent background noise level (Supplementary Fig. 13). Even for 8-h co-incubation experiments at 55 ℃, the detected Rhodamine 6G did not exceed 10^−7^ µM (lower than 10^−7^%) in FC-70 (Supplementary Fig. 13). Note that Rhodamine 6G is considered as the worst-case scenario for diffusion in a fluorinert (Novec 7500) in Gruner’s 2016 study^48^. These results support the claim that the FC-70 carrier oil is highly resistant to diffusion of reagents and, for the time scales associated with typical reaction matrices in microfluidics, is sufficient to prevent diffusion-induced cross-contamination. Therefore, this low risk of cross-contamination allows one to freely program a complex cascade of reactions with overlapping fluidic paths, especially when dealing with routine reagents that already have a low molecular diffusivity.

Furthermore, a Hela S3 cell suspension is used to evaluate the impact of acoustic streaming with a digital acoustofluidic device on cell viability. After 20 min of trapping, a propidium iodide (PI)-calcein AM (CAM) double staining assay indicated the viability was 99.2%, which has almost no difference when compared against the control group (99.9%, Supplementary Fig. 14).

### Liquid handling of enzymatic reagents with digital acoustofluidics

Enzymes play an essential role in catalyzing many biomedical reactions and accelerating routine detection and diagnostic protocols. However, with sensitive protein binding sites, enzymes are generally chemically sticky and sensitive to contamination. Therefore, a trace amount of a target protein or other interfering reagent adsorbed on the physical surface could lead to cross-contamination and unpredictable results in a reaction. In addition, due to their sticky nature, enzymes may undergo conformational changes or denaturation when in contact with a solid surface.

Herein we demonstrate the capabilities of digital acoustofluidics by performing enzymatic reactions because its non-contact advantage eliminates any surface adsorption. As a calibration standard, a well-established glucose detection experiment was chosen as a model to validate the use of floating droplets for enzymatic reactions. The colorimetric readings from glucose detection reactions on the digital acoustofluidic devices are correlated to the glucose concentration and are compared to that of the standard method, which uses bulk catalysis in a 96-well plate (Supplementary Fig. 15). The correlation (higher than 0.99) and linear regression coefficients (*R*^2^ is higher than 0.99 when the glucose concentration is higher than 0.01 mg ml^−1^, *R*^2^ is higher than 0.87 when the glucose concentration is <0.01 mg mL^−1^, Supplementary Fig. 16) of the digital acoustofluidic-based reactions match well with the standard method. This indicates that the floating droplets are reliable containers suitable for enzymatic reactions. Interestingly, the colorimetric readings in these floating reactors are generally greater than the standard reactions by 30%, which could imply that the reactions are faster due to the rapid mixing from the internal-streaming effect within the droplet.

**Fig. 7:**
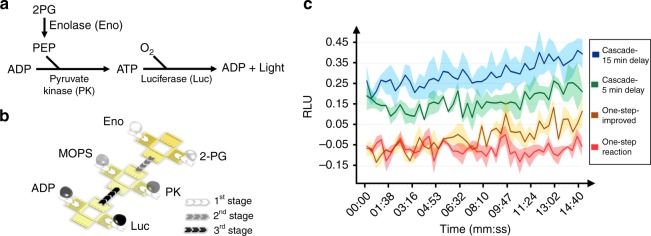
Enzyme cascade reaction for detecting enolase on a digital acoustofluidic device. **a** The mechanism of enolase detection. **b** Illustration of the six-droplet-merging sequence of the cascade reaction for enolase detection. **c** A comparison of the results for detecting 50 ng mL^−1^ of enolase with respect to elapsed time, performed using the one-step reaction on a plate and the cascade reaction using digital acoustofluidics. The relative luminescence units (RLU) are normalized to the negative control (no enolase) case. For the classic one-step reaction (red curve), the entire reaction mixture is applied to enolase sample immediately. In the improved one-step reaction configuration (yellow curve, labeled ‘One-step-improved’), the reaction mix is incubated for 5 min to wait for the background from 2-PG, ADP, Luc to fade before adding the enolase sample. For the cascade reactions, the enolase sample is firstly incubated with 2-PG for 5 min (green curve, labeled ‘Cascade-5 min delay’) or 15 min (blue curve, labeled ‘Cascade-15 min delay’) to produce an extra amount of PEP, which will then trigger a more intense signal increase with the other enzymes and substrates

In order to demonstrate the capability of the digital acoustofluidics to handle a cascade of reactions with multiple reagents, we selected the detection of neuron-specific enolase (NSE) as a proof-of-concept experiment with real-world applications. The glycolytic enzyme NSE is typically released from damaged neurons and has been suggested to be used as a biomarker for rapid diagnosis of various brain injuries (e.g., stroke and concussions) and for prognosis after brain surgery^49^. Generally, only a short window (i.e., 3–4 h) is available for the most effective treatments after the onset of brain damage, so a 15 min enzyme-based reaction will provide timely diagnostic evidence for deciding treatment options. As shown in Fig. 7a, a three-step coupled-cascade reaction is employed to detect the enolase: step 1, enolase catalyzes 2-phosphoglycerate (2-PG) to phosphoenolpyruvate (PEP); step 2, pyruvate kinase (PK) converts PEP and adenosine diphosphate (ADP) to pyruvate and adenosine triphosphate (ATP); and step 3, luciferase (Luc) consumes an ATP molecule to generate a photon which is detected.

For biochemists, a classic protocol for enolase detection is to apply a 5-reagent reaction mix (i.e., 2-PG, ADP, PK, Luc, and MOPS buffer) to enolase samples to generate a detectable luminescent signal. This one-step reaction is simple and accommodating to manual operations in clinical use. However, in the simple reaction mix, 2-PG and ADP produce a strong background with Luc, which obscures the signal originating from the enolase. At low concentrations (enolase lower than 100 ng mL^−1^), this protocol typically requires 40‒60 min to reveal the small luminescence difference between test groups and control groups. This delay may introduce false-negative results or critical delays in the urgent delivery of medical treatment.

Using digital acoustofluidics, multiple droplets with six different reagents are transported and merged following an optimized schedule (Fig. 7b). The 2-PG is incubated with enolase (50 ng mL^−1^) for 5‒15 min to produce an extra amount of PEP, which will then react with the other enzymes and substrates. This extra amount of PEP produces bonus ATP with ADP and PK, which subsequently triggers a more intense luminescence signal with Luc. In the classic one-step reaction, the Luc receives less ATP at any given time and hence the luminescence signal is weaker than in the case of the cascade reaction (Fig. 7c). This cascade configuration on the digital acoustofluidic device enhances the signal-to-noise ratio (SNR) by more than three times, improving the limit-of-detection, and makes 50 ng mL^−1^ enolase detectable at the beginning of the reaction, which significantly shortens the diagnosis cycle for acute stroke patients. Furthermore, since using digital acoustofluidics it is now not necessary to pipette samples from container to container, our platform is suitable for the scalable automation of routine fluidic processing tasks.

## Discussion

We have present digital acoustofluidics as a droplet handling and processing technique based on the use of acoustic streaming to allow reagent transport over shared, overlapping paths without cross-contamination. This is effectively a rewritable and programmable fluidic processor. In comparison to existing fluidic processing approaches, the digital acoustofluidics platform has at least four advantages:

Rewritability: The contactless liquid-handling mechanism inherently eliminates cross-contamination associated with surface adsorption and the need for surface modification. Since the aqueous droplets are isolated from the substrate by a fluorinated oil that is highly inert and immiscible, different reagents can float along the same path in any sequence of fluidic transport and mixing^47^. This feature enables reusable paths for the ‘fluidic inputs’ (i.e., droplets) to be dynamically processed on arbitrary routes without cross-talk between each other. It also enables unprecedented rewritability and scalability. This rewritability exponentially increases the allowable number of combinations of reagent inputs on the same device as the array dimensions, due to the number of independent inputs, and the levels of cascading layers of the reactions increases. For a fluidic processor with *N* × *N* pixels, a non-rewritable processor can only render less than *N*^2^ combinations for single-step reactions. Whereas in the digital acoustofluidic system, a rewritable processor can exploit all the possible combinations:_4*N*_*C*_*M*_, of arbitrary *M* agents among 4 *N* inputs on the same processor. For reactions with M arbitrary reagents among different chemical inputs in one step (i.e., 4 *N* types of chemicals for an *N* × *N* array with inputs on its four edges), the possible number of combinations *C* is calculated by the following equation:

1 $$_{4N}C_M = \left( {\begin{array}{*{20}{c}} {4N} \\ M \end{array}} \right) = \frac{{\left( {4N} \right)!}}{{\left( M \right)!\left( {4N - M} \right)!}}.$$

Furthermore, this strategy also eliminates the need for chemical or physical surface modifications and the surface does not degrade due to continuous contact with possibly reactive or sticky liquids (e.g., blood), which results in a durable fluidic processor for performing successive, time-consuming experiments involving a wide variety of samples and reagents.

Biocompatibility: The droplets, instead of being directly subjected to strong acoustic pressure or high electric fields, are manipulated gently by hydrodynamic forces in which the flow speed is comparable to vortexing via manual shaking (i.e., mm/s level). This biocompatible liquid-handling process maximizes the potential for biological samples to retain their native states and properties. A PI-CAM protocol was used with Hela S3 cells to demonstrate that acoustic-streaming-based manipulation mechanism had excellent biocompatibility with no statistically significant effect on the viability of cells. These aqueous droplets are also ideal containers for preserving fragile bio-specimens (e.g., protein crystals^50^) that are sensitive to a solid-liquid interface. Additionally, the carrier oil, which is oxygen-permeable and inert with a wide range of biological samples (e.g., blood, urine, saliva, and fecal), indicating that these biological samples can access oxygen and will not interact with the oil.

Versatility: This fluidic actuation mechanism does not require extra modifications or labels in the droplets, suggesting digital acoustofluidics is not restricted to fluids with specific acoustic, electrical, hydrodynamic, or magnetic properties. This versatility makes digital acoustofluidics suitable for handling a wide range of liquids, even for challenging fluids such as low-polarity fluids (e.g., organic solvents), sticky or viscous samples (e.g., blood and sputum), and solids (e.g., fecal samples).

Uniformity: Digital acoustofluidics generates strong internal fluidic streaming within the trapped droplets, thereby minimizing internal polarization in aqueous droplets and enabling efficient mixing of merged droplets.

Another promising result is that with the same digital acoustofluidics mechanism, sub-nanoliter droplets can be actuated using IDTs with smaller dimensions and lower driving voltages. For example, a smaller version of the IDT (i.e., Supplementary Fig. 9) requires a lower operating voltage (i.e., 5‒7 Vpp) to actuate droplets, which is promising for scaling-up and increasing the number of droplets that can be simultaneously controlled. This has a positive impact on the scalability potential of digital acoustofluidics in terms of large-scale integration, process parallelization, and high-throughput automation of micro-reactions with extremely low reagent consumption.

The present layout of electrical interconnections raises issues such as variations in wire impedances and overlapping wiring paths when scaling-up. The planar wiring layout or the continuity of the transducer surface is not mandatory for digital acoustofluidics. Since the droplets are manipulated in a contact-free manner, the transducers can be cross-connected three-dimensionally like a multilayer printed circuit board. The transducer array can be discretized into multiple individual transducer elements mounted on electric board (Supplementary Fig. 8), demonstrating the advantages of scalability due to multilayer connections, easy re-configurability, and uniform performance.

Unexpected evaporation can be suppressed by covering the droplet and carrier fluid with an extra oil layer (Supplementary Fig. 17), or via feedback control of the humidity similar to Poulikakos’ study^43^, which can culture cells in levitated droplets in air for 24 h. For droplet generation, the fluid reservoir (Supplementary Fig. 18) could be made of a disposable plastic material and be detachable from the IDT substrate underneath. The fluid reservoir can be designed on the same cartridge for product droplet recovery using a pipette and a hydrophobic oil absorber.

Active splitting of an aqueous droplet on a free surface of FC-oil is challenging based on the current digital acoustofluidics setup, due to the high surface tension of water (72 dynes cm^−1^, air) and the low surface tension of the carrier medium, FC-70 (18 dynes cm^−1^, air). The water droplets floating on FC-70 act more like an elastic sphere instead of a liquid, even with the addition of a Pluoronics surfactant to half the water’s surface tension. As shown in Supplementary Fig. 19, we demonstrate that in our digital acoustofluidic device, ethanol droplets (22 dynes cm^−1^, air) can be easily split on a silicone oil surface, due to its lower surface tension, using the two symmetric hydrodynamic traps that spontaneously form on both sides of the ascending jet.

In conclusion, we believe that digital acoustofluidics provides an excellent strategy for a durable, rewritable, and fully programmable fluid processor. It will significantly simplify liquid- handling and minimize protocol-routing bottlenecks in many biomedical applications such as automating enzymatic reactions, high-throughput aptamer-based systematic evolution of ligands by exponential enrichment (SELEX) screening, automated DNA/RNA sample preparation, drug testing, and programmable biomaterial synthesis.

## Methods

### Theoretical model

Detailed formulation, model description, force analysis, and parametric assessment can be found in the Supplementary Note 1. Digital modeling files are available upon reasonable request to Huang’s lab.

### Reagents and materials

The carrier oil is Fluorinert FC-70 (Hampton Research Corp., CA, USA). The viscosity of the carrier oil can be increased by dissolving Teflon® (AF1600, Dupont Co., DE, USA) to enhance the spatial stability of the floating droplets. The glucose detection kit, the SuperSignal® Femto-ELISA substrate, and the NSE detection kit are all from Sigma-Aldrich Corp. (Oakville, ON, USA).

### Software and electronics

The digital acoustofluidic device was powered with a 23.9 MHz sinusoidal AC signal from a function generator (DG 3012C, Teletronics Technology Corporation, PA, USA) and an amplifier (25A250A, Amplifier Research, USA). A relay array (USB24Mx, EasyDAQ, UK) was used to control the power input for individual transducers. The control program was written in Visual C++ (Microsoft Corp., USA).

### Device fabrication and operation

5 nm Cr/50 nm Au was deposited on 128^o^ Y-cut lithium niobate wafer (Precision Micro-Optics, USA) after standard photolithography. The electrical connections between the chip and external wires were made using silver epoxy (MG Chemicals, USA). A network analyzer was used to determine the optimal device operating frequency prior to operation. It varies from device to device (23.7‒24.5 MHz, 40.0‒57.2 Vpp) due to manual errors during fabrication (e.g., wafer-mask alignment). The dimensions of the transducer were 3.124 mm in length and 2.240 mm in width. The pitch distance between pixels was 3.124 mm. The layout for electric connections is shown in Supplementary Fig. 20. The 64 IDTs share the same ground plane and can be actuated by 64 individual signal pads.

### Sub-nanoliter droplet manipulation

The sub-nL droplet was generated via a conventional T-junction microfluidic chip. The generated droplets were guided to the digital acoustofluidic device through a capillary tube. The dimensions of the individual acoustic transducers were minimized to 1.1 × 0.8 mm. The transducer operated at 49.125 MHz, 5.0‒7.0 Vpp.

### Droplet generation

The fluid reservoir is 3D-printed in Rainbow Flexible plastic (J750 Stratasys, Ltd., USA). The IDT has identical dimensions and resonant frequency as the IDT unit shown in Fig. 1b but is excited at 24.5 MHz for oil jetting (excitation voltage: 300 Vpp, duration, 30 ms). An IDT substrate was attached to at the bottom of fluid reservoir and its geometric center was aligned with the center of nozzle. A hydrophobic ring made of hot-melt adhesive is attached to the nozzle (diameter: 2 mm) as a surface-tension barrier to prevent the aqueous solution from escaping spontaneously due to buoyancy.

### Droplet splitting

The droplet volume was 25 µL. A total of 2.7 mm thick FC-70 oil layer was used to isolate the substrate and the floating droplets. The IDT was excited for 150 ms at 150 Vpp, 23.9 MHz for droplet splitting.

### Diffusion study

The fluorescein and rhodamine 6G were dissolved in pure water to a final concentration of 100 µM. Quantity of 500 µL of dye solution was dispensed with 700 µL of carrier oil into a 96-well plate for incubation. The incubation tests had different elapsed times (0.5 h, 1 h, 2 h, 4 h, and 8 h) and environment temperatures (20 ℃, 30 ℃, and 55 ℃). After incubation, 500 µL of carrier oil was carefully transferred from each well to a new plate for fluorescence measurement by a plate reader (480 nm excitation/500 nm emission, Synergy HT, BioTek Instruments, Inc., USA).

### Viability study

A Quantity of 50 µL of Hela S3 cells (density of 10^6^ cell mL^−1^) in a suspension was dispensed onto the carrier oil and trapped by an activated IDT. The cells were double-stained with calcein AM (CAM) and propidium iodide (PI) after the 20 min incubation. The viability was measured by counting CAM-positive and PI-negative cells using flow cytometry (FC500, Beckman Coulter, Inc., USA).

### Glucose detection experiments

Six droplets, each with a volume of 30 µL, with different glucose concentrations (0 mg mL^−1^, 0.0005 mg mL^−1^, 0.001 mg mL^−1^, 0.01 mg mL^−1^, 0.03 mg mL^−1^, and 0.05 mg mL^−1^) were first sorted into six separate digital acoustofluidic traps, and then were separately, sequentially merged with a 20 µL reaction mix (containing ODD, MOPS buffer, and HRP). A positive control was run on a round-bottomed 96-well plate. The temperature of the plate-based reaction was calibrated to be the same as the floating droplets at room temperature by using a Peltier plate. After reacting for 20 min, 40 µL of the product from this colorimetric reaction was transferred to the round-bottomed 96-well plate for detection by a plate reader (425 nm, Synergy HT, BioTek Instruments, Inc., USA). During the reaction, hydrogen peroxide was generated upon oxidization with glucose oxidase (GOx) and FAD/FADH_2_ mediated electron transfer. The reporter molecule o-dianisidine dihydrochloride (ODD) was subsequently oxidized by the hydrogen peroxide via the HRP catalyst and this produced a colorimetric (optical absorbance) change that can be used to quantify glucose concentrations.

### Neuron-specific enolase (NSE) detection experiments

2-phosphoglycerate (2-PG) barium salt, rabbit pyruvate kinase (PK), adenosine diphosphate (ADP), 3-morpholinopropane-1-sulfonic acid (MOPS), and enolase were purchased from Sigma-Aldrich Corp., USA. The barium in 2-PG needed to be replaced with sodium to become soluble in water. The ATP luciferase assay kit was purchased from Promega Corp., USA. In order to minimize unwanted electromagnetic interference effects from the 23.9 MHz actuation signal, a quasi-simultaneous strategy was implemented via the controlling software to alternatively excite multiple IDTs to maintain and control the positions of 6 droplets. 1^st^ stage reaction: an enolase-droplet (50 ng mL^−1^, 10 μL) was merged and mixed with a 2-PG-droplet (30 mM, 10 μL) and is labeled as the Product Droplet 1; a MOPS-droplet (10 μL) was merged with a PK-droplet (20 unit mL^−1^, 5 μL) and labeled as Product Droplet 2; an ADP-droplet (60 mM, 5 μL) was merged with a luciferase-droplet (10 μL) and labeled Product Droplet 3. 2^nd^ stage reaction: Product Droplet 1 (20 μL) merged with Product Droplet 2 (10 μL) to produce Product Droplet 4 which was incubated for 5‒15 min to minimize the background fluorescence. 3^rd^ stage reaction: Product Droplet 3 (20 μL) was merged and mixed with Product Droplet 4 (30 μL) to produce the final reaction product. The liquid handling process is demonstrated in Supplementary Fig. 21. A quantity of 45 μL of the final reaction product was transferred from the chip to the plate reader (gain: 200, integration time: 1 s, detector height: 1 mm) by pipette for luminescence detection after 15 min. The luminescence values were then normalized with respect to the control group and then plotted.

### Data availability

The data supporting the findings in this paper are available from the corresponding author upon reasonable request.

## Electronic supplementary material

Supplementary Information

Description of Additional Supplementary Files

Supplementary Movie 1

Supplementary Movie 2

Supplementary Movie 3

Supplementary Movie 4

Supplementary Movie 5

Supplementary Movie 6

Supplementary Movie 7
