# Supplementary material for: Digital acoustofluidics enables contactless and programmable liquid handling
Source: Nat Commun. 2018 Jul 26;9:2928. doi: 10.1038/s41467-018-05297-z (PMC6062562; doi:10.1038/s41467-018-05297-z)
Supplement: Supplementary file 1 — Supplementary Information [file 41467_2018_5297_MOESM1_ESM.pdf]

Supplementary Information

Digital Acoustofluidics Enables Contactless and Programmable Liquid Handling

Steven Peiran Zhang[a], James Lata[b], Chuyi Chen[a], John Mai[c], Feng Guo[b], Zhenhua Tian[a], Liqiang Ren[b], Zhangming Mao[b], Po-Hsun Huang[a], Peng Li[b], Shujie Yang[a], and Tony Jun Huang[a,1]

[a] Department of Mechanical Engineering and Material Science, Duke University, NC 27708, USA;

[b] Department of Engineering Science and Mechanics, The Pennsylvania State University, University Park, PA 16802, USA;

[c] Alfred E. Mann Institute for Biomedical Engineering, University of Southern California, Los Angeles, CA 90007

[1] To whom correspondence should be addressed. Email: tony.huang@duke.edu

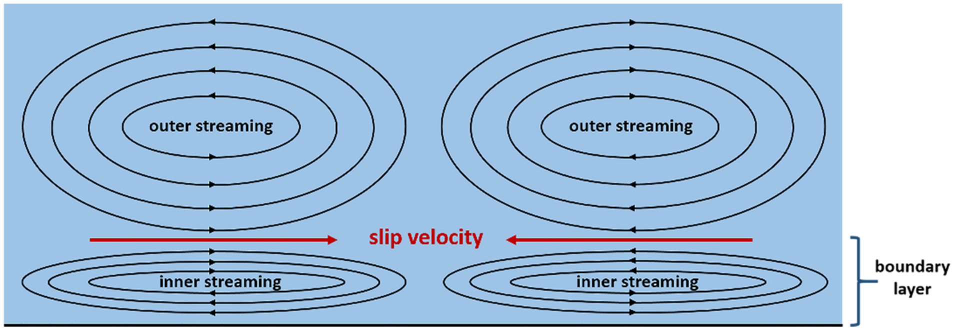

Supplementary Figure 1. Illustration of boundary-layer-driven streaming in the z-plane.

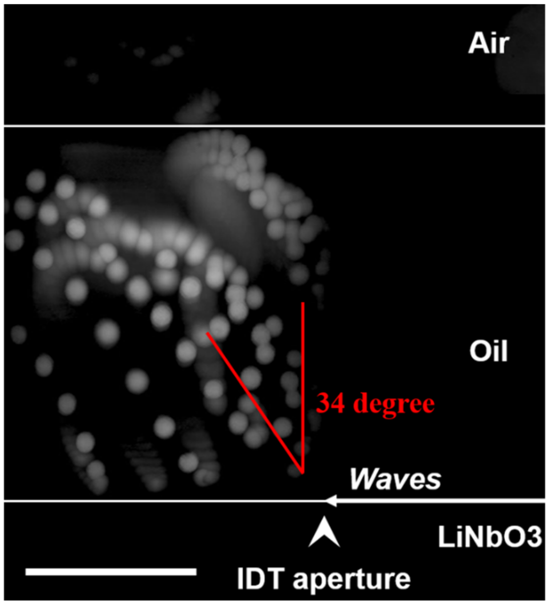

Supplementary Figure 2. Side view of the IDT aperture. The jetting streamlines within the oil layer are visualized using 105 µm diameter fluorescent particles and a small prism. The jetting angle at the IDT aperture is 34 degrees. The scale bar is 1 mm.

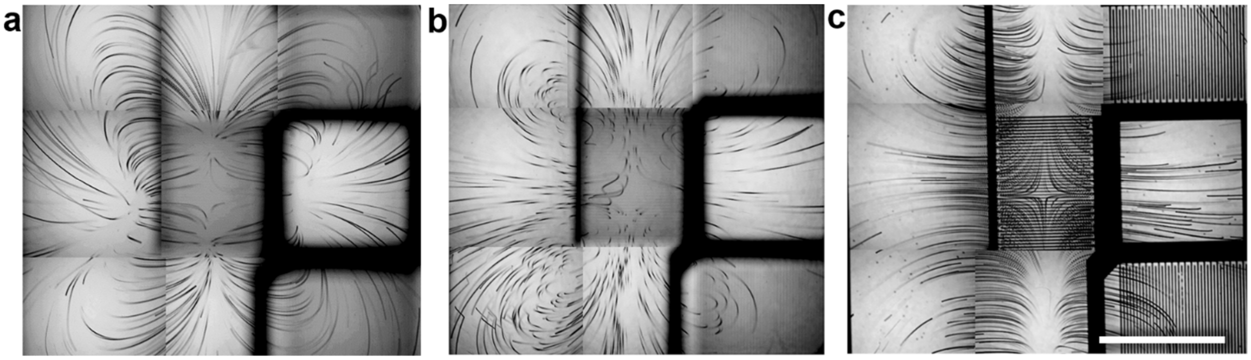

Supplementary Figure 3. Particle trajectories on the top surface plane of the oil (a) (z = 2 mm), in the middle of the oil (b) (z = 1.3 mm), and at the bottom plane of the oil (c) (z = 0 mm). Each composite image consists of 9 independent images that are spatially aligned. The excited IDT is located at the center of the composite image. The scale bar is 3 mm.

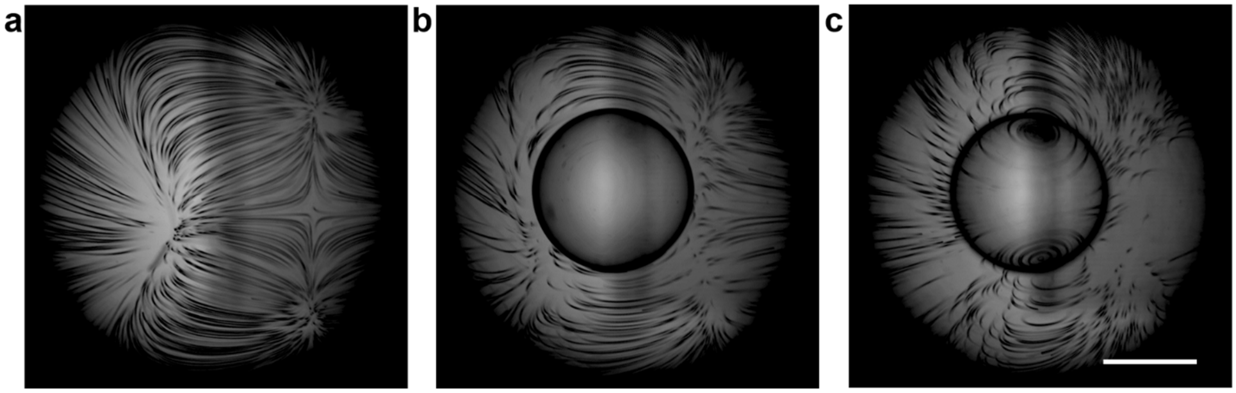

Supplementary Figure 4. Streaming pattern. (a) Top view of the streaming patterns of a hydrodynamic trap. The focal plane of the microscope is at the surface of the oil. (b) Once the trap is filled with a droplet, the streaming patterns changed spontaneously but retains a similar pattern. The microscope imaging focal plane is on the surface of oil. (c) The streaming patterns of a hydrodynamic trap that is filled with a droplet. The focal plane is now 0.5 mm below the oil surface. The scale bar is 1 mm.

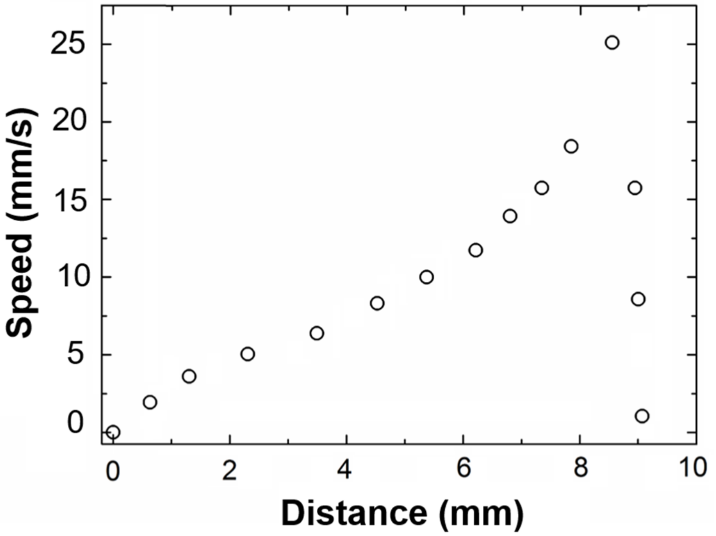

Supplementary Figure 5. A graph showing the acceleration of a droplet as it traverses a fixed step distance of 9 mm, as a function of droplet speed for evenly spaced elapsed-time intervals. The droplet gradually accelerates in the first 8.5 mm and drastically decelerates in the final 0.5 mm.

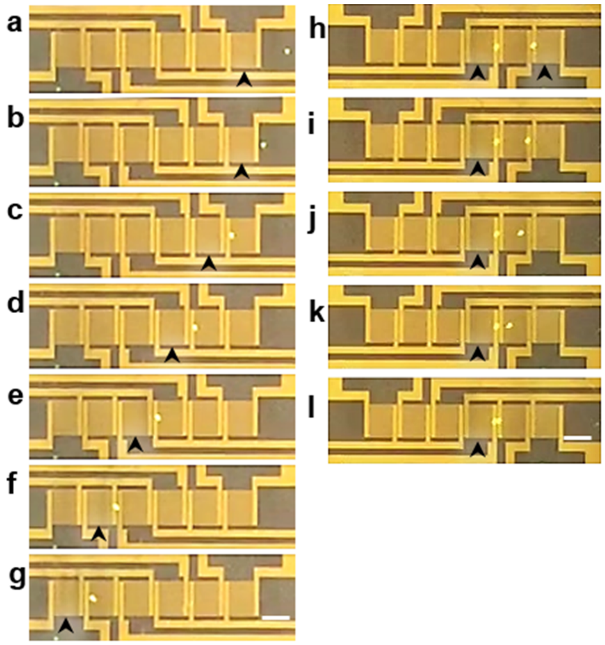

Supplementary Figure 6. Micro-particle manipulation using digital acoustofluidics. (a) – (g) Single-particle manipulation using digital acoustofluidics. The device configuration is the same as for sub-nanoliter droplet manipulation (Supplemental Figure 8). (h) – (i) Particle pairing using digital acoustofluidics. The particle density is 1.1 g·cm^{-3} and the diameter is 105 µm. The black arrows indicate the excited transducer. The scale bar is 0.7 mm.

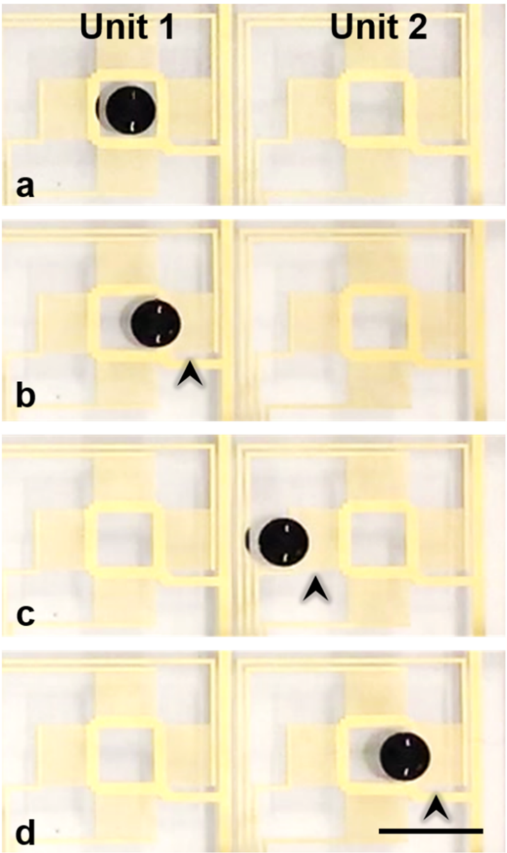

Supplementary Figure 7. A trap-and-release mechanism from pixel to pixel (Unit 1 to Unit 2) (a) using an off-chip electromechanical relay controller located on a separate printed circuit board. The black arrow indicates the activated transducer. The droplet was first moved and trapped by the IDT on the right side of pixel-unit 1 as indicated by the black arrow (b). Immediately after the droplet was released when the IDT on the right side of pixel-unit 1 was turned off, the IDT on the left side of pixel-unit 2 was excited and the droplet was continuously translated and trapped by the excited IDT (c). Following a similar relaying principle, the droplet was finally trapped by the IDT on the right side of pixel-unit 2 (d), and transport of the droplet from pixel-unit 1 to pixel-unit 2 was completed. The scale bar is 6 mm.

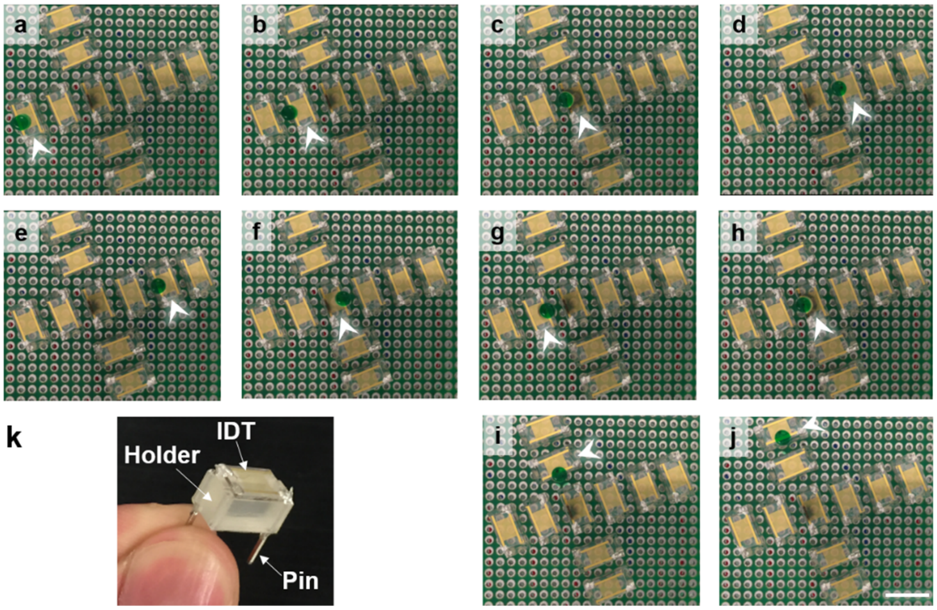

Supplementary Figure 8. Contactfree droplet transportation using reconfigurable transducer array. (a) – (j) Droplet translation using discrete transducers on a printed circuit board which provides electrical connectivity. Scale bar: 10 mm. (k) A picture of a discrete transducer on an adaptor.

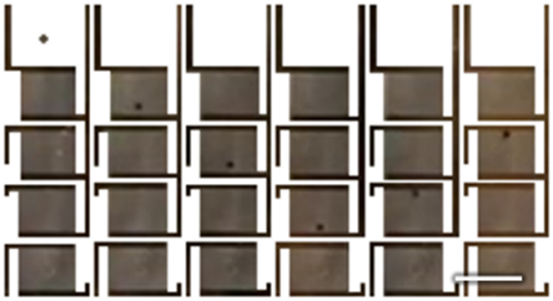

Supplementary Figure 9. Sub-nanoliter droplet manipulation using digital acoustofluidics. The operating parameters for the IDTs (*e.g.*, frequency, dimensions, and spacing) are optimized. The droplets are generated using a T-junction in a conventional microfluidic chip and then are re-injected onto the carrier oil. The measured volume of the droplet is 624 pL. The scale bar is 0.8 mm.

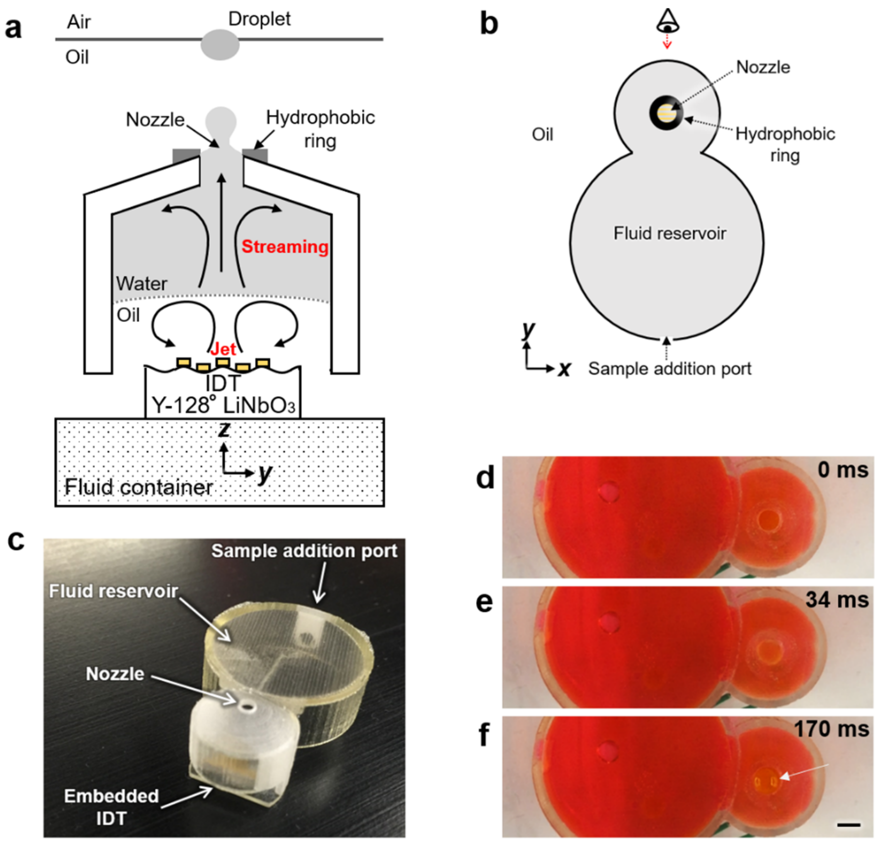

Supplementary Figure 10. Acoustofluidic-based on-demand droplet generation *via* acoustic streaming. (a) Schematic showing a y-z-plane cross-sectional view of the mechanism for acoustofluidic-based droplet generation. With acoustic streaming, a droplet can be generated on-demand by squeezing the aqueous solution out of the nozzle of a fluid reservoir embedded beneath the surface of oil layer. The IDT at the bottom of reservoir generates acoustic waves and jets oil upward, thereby impinging on and pushing the aqueous solution through the nozzle, which serves as a surface tension barrier to restrict spontaneous flow from buoyancy (Supplementary Movie 6). (b) A top view schematic of the acoustofluidic droplet generator. The structures in schematic A are aligned underneath the nozzle in this top view. Aqueous sample is added through the sample addition port on the side-wall of fluid reservoir. (c) A photograph of the acoustofluidic droplet generator device without any working fluids. (d) – (f) Time-lapsed image capture of acoustofluidic droplet generation from Supplementary Video 6. The IDT underneath was excited at 300 Vpp for 30 ms to generate one droplet. A fluid jet can be seen in the nozzle region in image-D2. The white arrow in image-D3 indicates the position of the generated droplet, which is now floating on the oil surface. Scale bars: 2 mm.

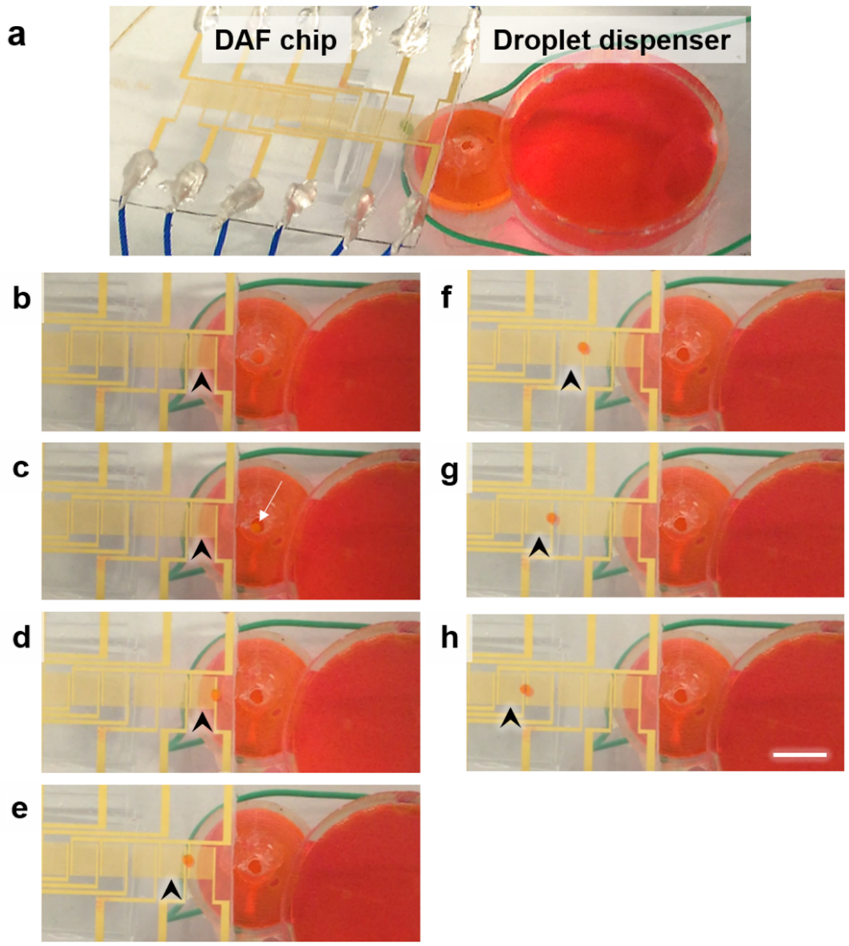

Supplementary Figure 11. Interface between a digital acoustofluidic chip and a droplet generator. (a) Photographs showing the integration of the acoustofluidic droplet generator and the digital acoustofluidic (DAF) chip. Both the DAF chip and the droplet generator are embedded beneath the oil layer. The nozzle is aligned with a hydrodynamic trap on the DAF chip. (b) – (h) Droplet generation and transportation on the DAF chip. The white arrow in image B2 indicates the position of the generated droplet. Scale bar: 5 mm.

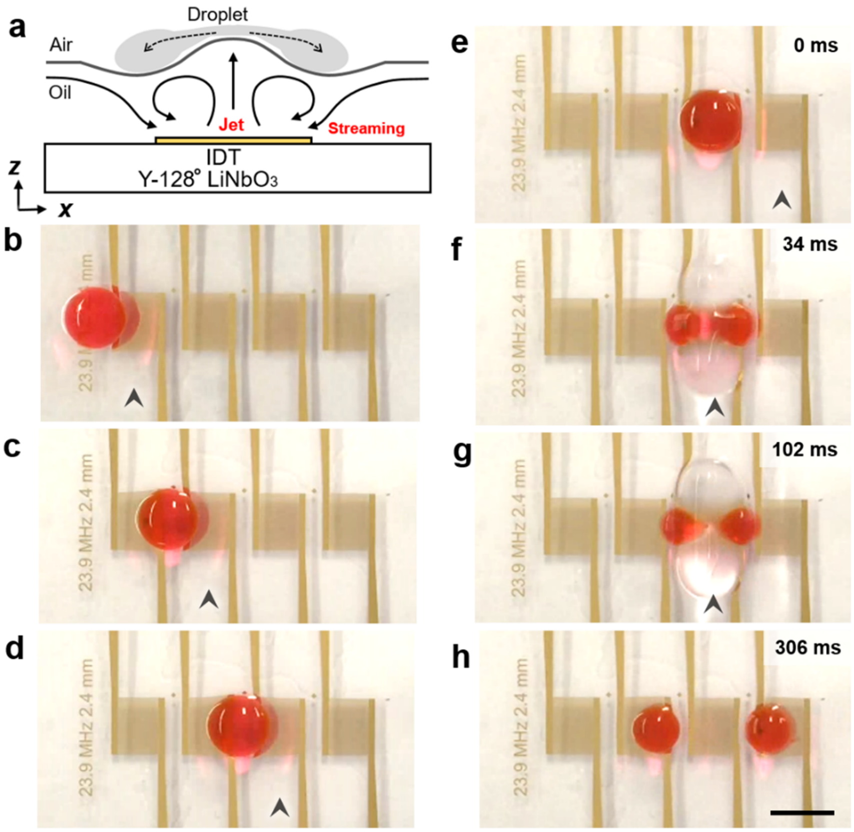

Supplementary Figure 12. Active splitting of a water droplet on FC-70. (a) Schematic *x*-*z*-plane cross-sectional view of the streaming pattern at a transducer. Droplet transportation process (b) – (d) and on-demand splitting process (e) – (h). Excitation voltage for splitting: 150 Vpp. Duration: 150 ms. Scale bar: 5 mm.

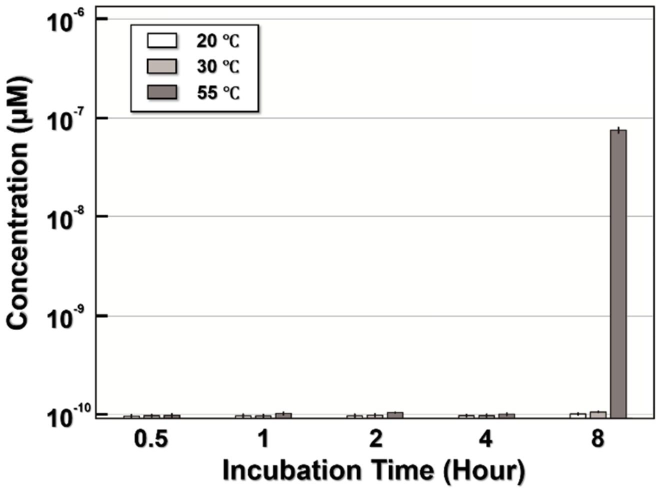

Supplementary Figure 13. Characterization of diffusion into the carrier oil. Rhodamine 6G (100 μM, 500 μL) is used to characterize the diffusion.The dye solution is co-incubated with a fluorinert oil for different incubation times (0.5, 1, 2, 4, and 8 hrs) and at different temperatures (20, 30, and 55 ^{o}C). After incubation, 200 μL of oil is carefully transferred to a new plate for fluorescence detection. The procedure is detailed in the '**Methods**' section. The diffusion of rhodamine 6G is below the limit of detection (lower than 10^{-10} μM) in all co-incubation experiments at a typical range of room temperatures (20 ~ 30 ^{o}C).

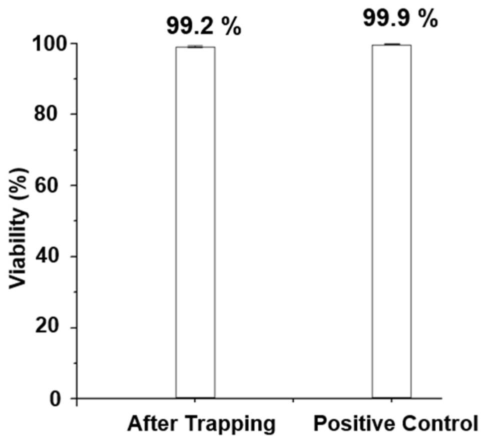

Supplementary Figure 14. Cell viability test. A 50 μL droplet of Hela S3 cell suspension (10^6 cell·mL^{-1}) is trapped and mixed for 20 min by a single IDT. Then the cell suspension is double-stained by CAM and PI to determine viability via flow cytometry (Beckman, FC 500). The counting result indicates there is not any considerable viability loss in the cells after being trapped and mixed on a digital acoustofluidic device for 20 min.

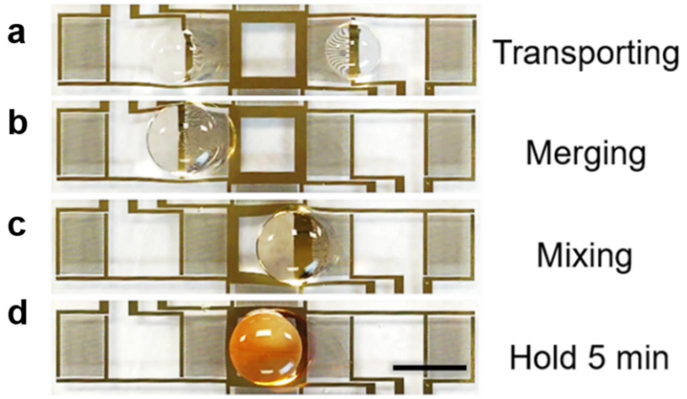

Supplementary Figure 15. Glucose detection reaction on a digital acoustofluidic device. A 1 M glucose droplet (30 μL) and a droplet containing a HRP reaction mixture (20 μL) are merged and mixed by sequentially activating the transducers beneath the oil (a) – (c). The product droplet is held for 5 min afterwards on chip to ensure a complete reaction (d). The scale bar is 3 mm.

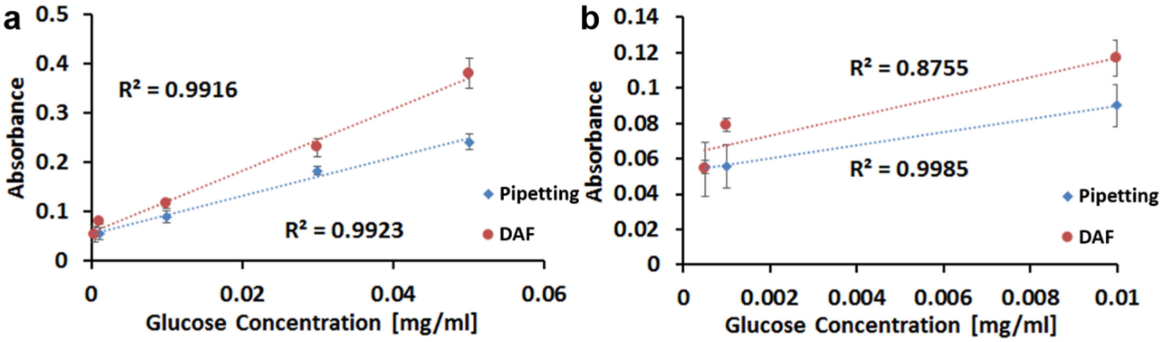

Supplementary Figure 16. Comparison of glucose detection between (a) the digital acoustofluidic device and (b) the standard pipetting method. The predetermined glucose concentrations are within the physiological concentration ranges in human saliva, urine, and serum. The linearity coefficient and determination coefficient are both greater than 0.99, which indicates the floating droplets can accurately mediate enzymatic reactions. However, the determination coefficient of the digital acoustofluidic platform (R^2 is higher than 0.87) is not as good as the plate when the glucose concentration is less than 0.01 mg·mL^{-1}.

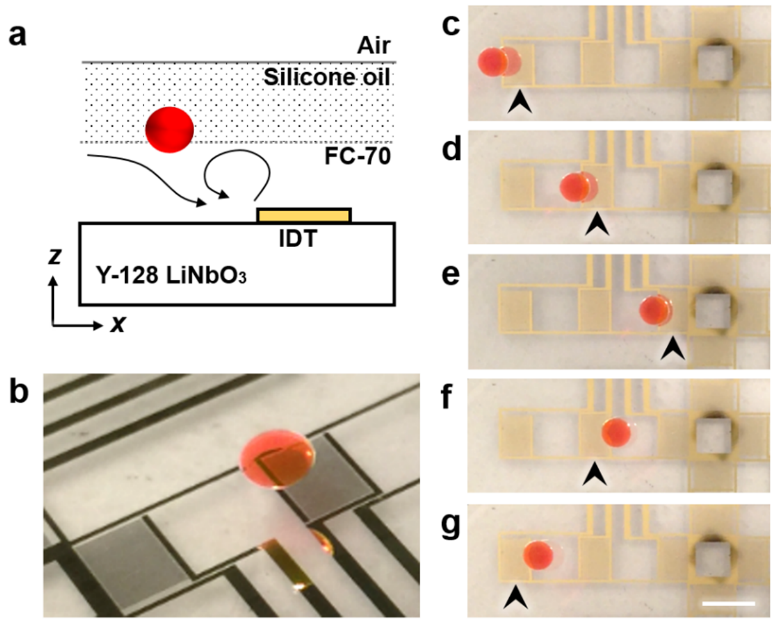

Supplementary Figure 17. Droplet manipulation with minimal evaporation. (a) Schematic cross-sectional view of droplet trapping at the interface between FC-oil and silicone oil (density: 0.913 g·cm^{-3}, viscosity: 5 cSt at 25°C). (b) A picture showing a trapped floating droplet covered with silicone oil. (c) – (g) Top view of droplet actuation between three IDTs with silicone oil layer. Scale bar: 5 mm.

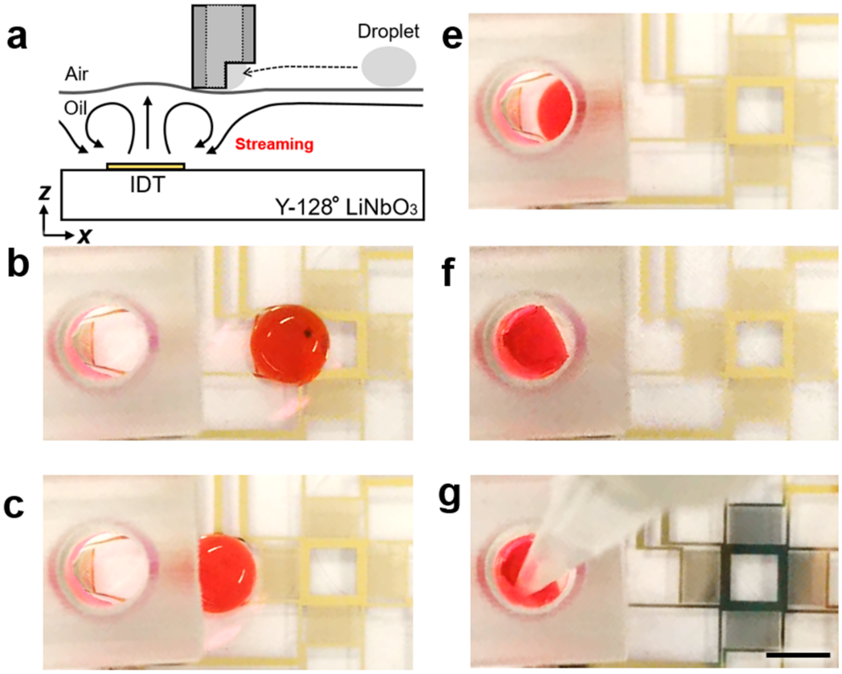

Supplementary Figure 18. Droplet collection via pipetting. (a) Schematic *x*-*z*-plane cross-sectional view of the droplet collector. (b) – (g) The droplet transportation and collection process. Scale bar: 5 mm.

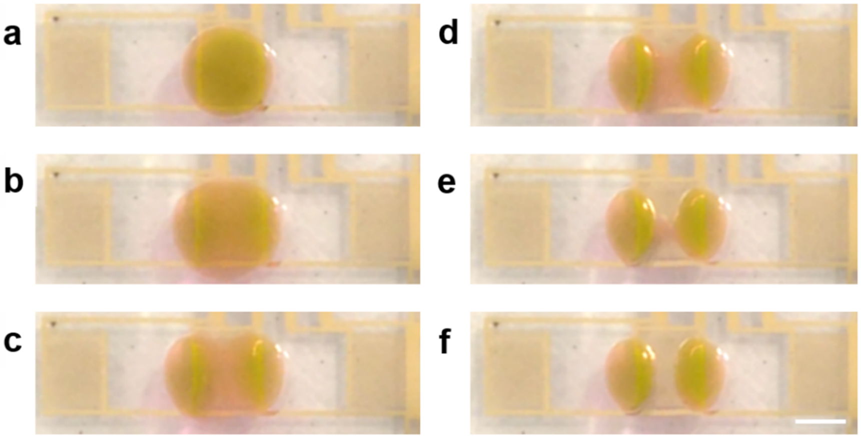

Supplementary Figure 19. Active splitting of an ethanol droplet on the surface of silicone oil using the digital acoustofluidic platform. Viscosity of silicone oil: 50 cSt. Scale bars: 4 mm.

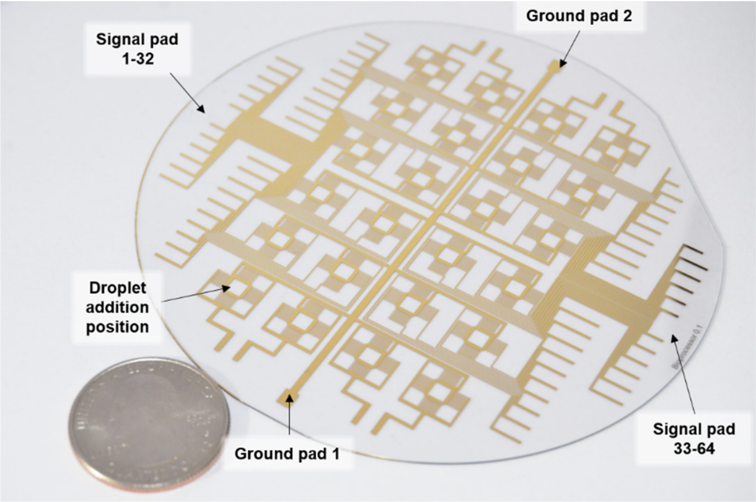

Supplementary Figure 20. Electric layout of the digital acoustofluidic chip shown in Fig. 1b.

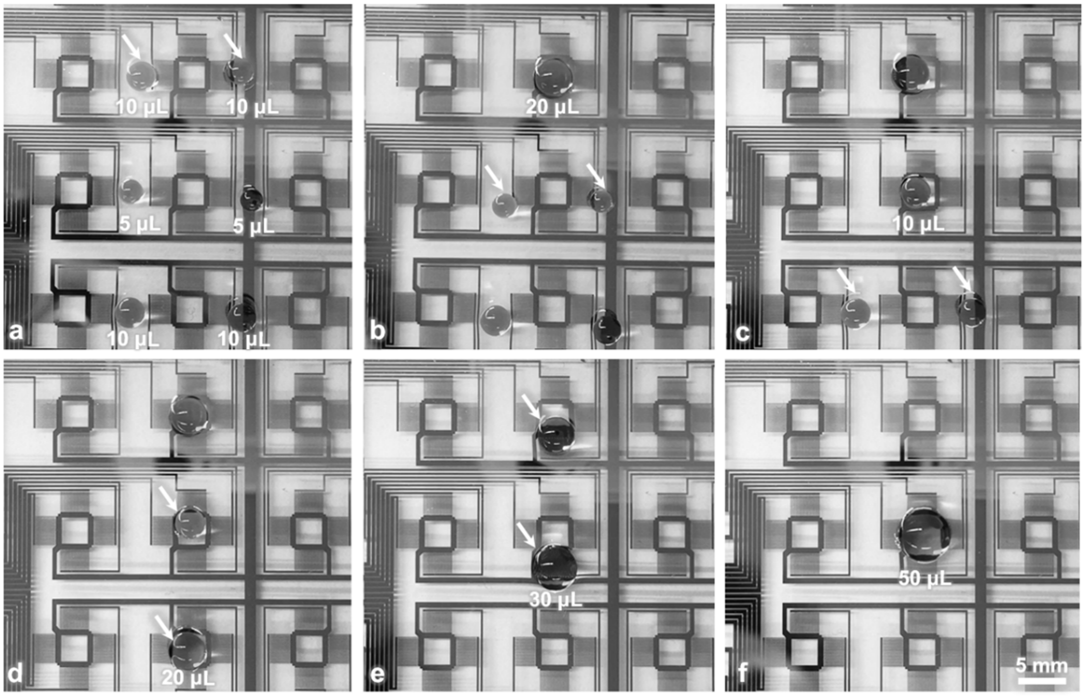

Supplementary Figure 21. Demonstration of the six-droplet cascade reaction with a digital acoustofluidic device (a) – (f). The white arrows indicate the droplet to be merged. Scale bar: 5 mm.

Supplementary Note1.

Theoretical models: The acoustic streaming, driven by the surface acoustic waves (SAWs) as they propagate from the IDTs into the fluidic layer is a type of boundary-driven acoustic streaming.[1,2] In order to simplify the numerical calculations and to minimize computational time, the acoustic streaming is modeled by defining a slip velocity, which is the velocity on the top of the inner streaming structure induced by SAWs within the boundary layer of the fluorinated oil. Hence, outside of the boundary layer, the Navier-Stokes equations and the continuity equation are solved by applying the slip velocity as the boundary condition at the bottom of the outer streaming field. The Navier-Stokes equations and continuity equation are:

$$\frac{\partial \rho}{\partial t} + \nabla \cdot (\rho \mathbf{v}) = 0 \,, \qquad [1a]$$

$$\rho \frac{\partial \mathbf{v}}{\partial t} = -\nabla p - \rho(\mathbf{v} \cdot \nabla)\mathbf{v} + \mu \nabla^2 \mathbf{v} + \left(\frac{1}{3}\mu + \mu_B\right) \nabla(\nabla \cdot \mathbf{v}) \,, \qquad [1b]$$

where ρ, μ and μ_B are the density, dynamic viscosity, and bulk viscosity of fluid, respectively, and p and $\mathbf{v}$ are the velocity and pressure in the fluid.

The slip velocity is determined by solving the first-order and second-order continuity and Navier-Stokes equations within the boundary layer area. The first-order and time-averaged second-order equations are:

$$\frac{\partial \rho_1}{\partial t} + \rho_0 \nabla \cdot (\mathbf{v_1}) = 0, \qquad [2a]$$

$$\rho_0 \frac{\partial \mathbf{v_1}}{\partial t} = -c_0^2 \nabla \rho_1 + \mu \nabla^2 \mathbf{v_1} + \left(\frac{1}{3}\mu + \mu_B\right) \nabla(\nabla \cdot \mathbf{v_1}), \qquad [2b]$$

$$\rho_0 \nabla \cdot \langle \mathbf{v_2} \rangle = -\nabla \cdot \langle \rho_1 \mathbf{v_1} \rangle, \qquad [3a]$$

$$-\nabla \langle p_2 \rangle + \mu \nabla^2 \langle \mathbf{v_2} \rangle + \left(\frac{1}{3}\mu + \mu_B\right) \nabla(\nabla \cdot \langle \mathbf{v_2} \rangle) = \rho_0 \langle \frac{\partial \mathbf{v_1}}{\partial t} \rangle + \rho_0 \langle (\mathbf{v_1} \cdot \nabla) \mathbf{v_1} \rangle \,. \qquad [3b]$$

The details of the derivation of these equations are explained in Ref. 3. The second-order velocity $(\mathbf{v_2})$ on the top of boundary layer is defined herein as the slip velocity.

Based on this model, the finite element method-based software package COMSOL 5.2a was used for numerical simulations. Within COMSOL, the “piezoelectric devices” modeling interface was used to model the surface vibration of the $LiNbO_3$ substrate that provides the activation energy to the fluid field. The “thermoviscous acoustics” and the “laminar flow” modeling interfaces solve for the physics within the boundary layer, whereas another “laminar flow” interface solves the physics without the boundary layer. The numerical interface between the substrate and the oil layer were coupled by the “thermoviscous acoustic-structure boundary”

condition in the COMSOL Multiphysics module. Taking advantage of symmetry, only a quarter of the fluid domain atop a quarter of a single IDT is modeled. The dimensions of the fluid domain model is 4 mm × 400 mm × 2 mm (W × L × H). The region with SAW propagation within the IDTs area at the bottom is 1.1 mm × 1.6 mm. As for the other boundaries, a symmetrical plane and wall boundary conditions are defined. The parameters used in the calculation are given as follows: f (frequency), 24 MHz; c, 640 m·s^{-1}; ρ_0, 1940 kg·m^{-3}; μ, 0.024 Pa·s.

Supplementary References:

1. Manor, O. *et al.* The appearance of boundary layers and drift flows due to high-frequency surface waves. *J. Fluid Mech.* **707**, 482–495 (2012).
2. Guo, F. *et al.* Three-dimensional manipulation of single cells using surface acoustic waves. *Proc. Natl. Acad. Sci.* **113**, 1522–1527 (2016).
3. Friend, J. *et al.* Microscale acoustofluidics: Microfluidics driven via acoustics and ultrasonics. *Rev. Mod. Phys.* **83**, 647 (2011).
