# Supplementary material for: Digital acoustofluidics enables contactless and programmable liquid handling
Source: Nat Commun. 2018 Jul 26;9:2928. doi: 10.1038/s41467-018-05297-z (PMC6062562; doi:10.1038/s41467-018-05297-z)
Supplement: Supplementary file 2 — Description of Additional Supplementary Files [file 41467_2018_5297_MOESM2_ESM.pdf]

Description of Additional Supplementary Files

File Name: Supplementary Movie 1
Description: **Inner streaming visualized by luminescence.** The transducer is turned on and off periodically to enhance mixing. Scale bar: 8 mm.

File Name: Supplementary Movie 2
Description: **The droplet-merging process.** Scale bar: 4 mm.

File Name: Supplementary Movie 3
Description: **Digital manipulation of a 65 nL droplet.** The working frequency is 104 MHz. The dimensions of the IDT are 400 µm × 600 µm. The liquid surface shakes slightly due to the vibration from the switching of IDTs. Scale bar: 2 mm.

File Name: Supplementary Movie 4
Description: **Cyclic transportation of a nanoliter-scale droplet.** The IDT switching frequency is 1 Hz.

File Name: Supplementary Movie 5
Description: **Cyclic transportation of a nanoliter-scale droplet.** The IDT switching frequency is 3 Hz.

File Name: Supplementary Movie 6
Description: **On-demand droplet generation.** The IDT excitation frequency is 1 Hz.

File Name: Supplementary Movie 7
Description: **On-demand splitting of a 25 µL droplet.** Scale bar: 5 mm.
